# GTP cycling protein (GCP): An ancient player for allosteric gatekeeping

**DOI:** 10.1002/mlf2.70043

**Published:** 2025-10-22

**Authors:** Ji‐Long Liu

**Affiliations:** ^1^ School of Life Science and Technology, ShanghaiTech University Shanghai China; ^2^ Department of Physiology Anatomy and Genetics, University of Oxford Oxford UK

Guanosine triphosphate (GTP), a purine nucleotide central to cellular function, has long been celebrated as both an energy currency and a dynamic regulator of life's most fundamental processes^1^. Its canonical role as an energy carrier—donating phosphate groups to drive reactions ranging from protein synthesis to microtubule polymerization—has been extensively characterized, paralleling the functions of its more abundant counterpart, adenosine triphosphate (ATP)^2^. Yet, GTP's regulatory versatility extends far beyond energy transfer. As a signaling molecule, GTP orchestrates critical cellular responses, from neurotransmission to immune activation, by binding and modulating the activity of G‐protein‐coupled receptors (GPCRs) and small GTPases such as Ras, Rab, and Gα subunits^3^. These canonical GTPases rely on GTP hydrolysis to trigger conformational changes, enabling transient signal amplification before terminating cascades through GTP‐to‐GDP conversion.

However, emerging structural and mechanistic insights challenge this binary view of GTP as merely a “fuel” or “switch”. Recent studies reveal a distinct class of GTP‐binding proteins that utilize non‐hydrolytic GTP cycling—iterative binding and dissociation without phosphate bond cleavage—to gate enzymatic activity^4^, ^5^ (Figure 1). This study introduces GTP cycling proteins (GCPs), a conserved family of regulators that redefine metabolic efficiency and allostery. An example is the rate‐limiting enzyme in de novo cytidine triphosphate (CTP) biosynthesis CTP synthase (CTPS). Unlike hydrolytic GTPases, CTPS employs GTP binding as a stoichiometric timer, coupling nucleotide availability to biosynthetic output through three sequential checkpoints: substrate entry primed by GTP dissociation, intermediate‐dependent GTP rebinding, and ammonia tunnel assembly^4^, ^5^. This mechanism not only preserves energy under stress but also enables precise metabolic flux control, positioning GCPs as ancient sentinels of cellular resilience.

**Figure 1:**
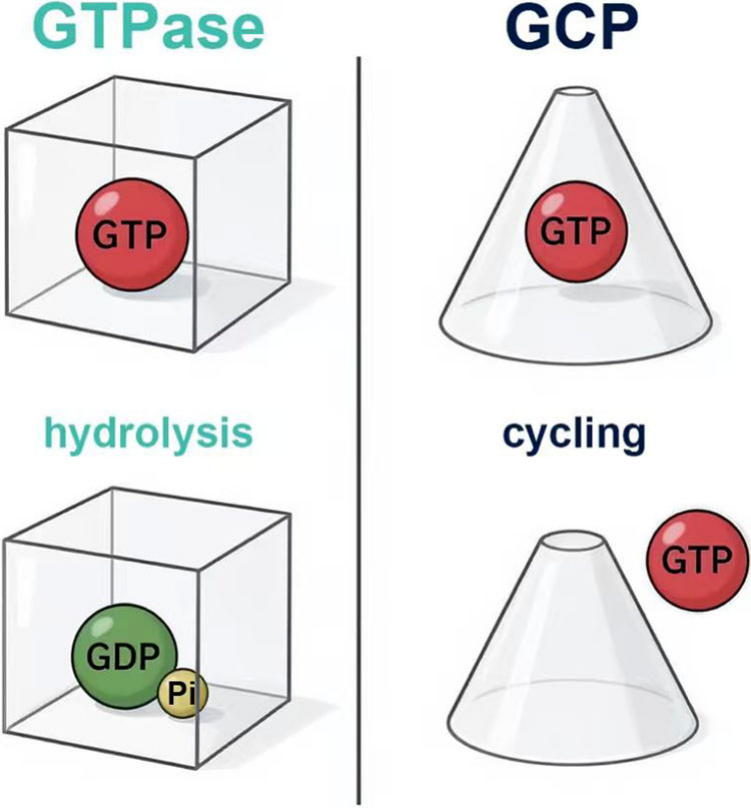
GTPase versus GTP cycling protein (GCP). While classical GTPases rely on GTP‐to‐GDP hydrolysis (left panel: hydrolysis cycle), GCPs utilize non‐hydrolytic GTP binding‐dissociation cycling (right panel: cycling process). Transparent cube: GTPase (undergoes conformational changes linked to GTP hydrolysis, converting GTP to GDP + Pi). Transparent cone: GCP (engages in GTP binding and release without hydrolysis, maintaining GTP‐bound states through cycling). Red ball: GTP (substrate for both GTPase hydrolysis and GCP binding). Green ball: GDP (hydrolysis product of GTPase, not involved in GCP cycling). Yellow ball: Pi (inorganic phosphate, released by GTPase hydrolysis, absent in GCP processes).

Here, I synthesize these advances to propose GCPs as a unified framework for understanding non‐hydrolytic regulation. By elucidating their ancient origins, mechanistic versatility, and therapeutic relevance, I redefine GTP's role beyond energy currency, unveiling a dynamic model for metabolic and allostery control.

## CTPS AS A MODEL FOR GTP CYCLING: STRUCTURAL INSIGHTS FROM CRYO‐ELECTRON MICROSCOPY (CRYO‐EM)

CTPS, the rate‐limiting enzyme in de novo CTP biosynthesis, exemplifies the GCP paradigm through its intricate interplay of non‐hydrolytic GTP binding and catalytic regulation^5^, ^6^. Notably, CTPS forms cytoophidia—elongated, filamentous structures—in vivo, which sequester and recycle nucleotide triphosphates to sustain biosynthesis^7^, ^8^, ^9^, ^10^, ^11^. Unlike canonical GTPases that dissipate energy via hydrolysis, CTPS employs iterative GTP binding/dissociation to orchestrate a catalytic cycle^4^. In a study which is pending peer review, cryo‐EM analyses of 34 conformational states (2.0–3.3 Å resolution) reveal three sequential checkpoints governed by GTP dynamics, each governed by distinct structural rearrangements^4^.

### Catalytic initiation gate

GTP dissociation precedes substrate entry, priming CTPS for UTP and glutamine binding. Structural data show that GTP binding induces a closed conformation of the glutamine amidotransferase (GAT) domain, preventing premature glutamine hydrolysis. GTP dissociation shifts conformation, creating a substrate‐accessible cavity.

### Intermediate‐dependent GTP recruitment

Formation of the 4Pi‐UTP intermediate (a linear uridine triphosphate intermediate) triggers allosteric remodeling of the GAT domain. The intermediate binds to a hydrophobic pocket, stabilizing a high‐affinity GTP‐binding site at the tetramer interface.

### Ammonia transfer gate

GTP directly participates in assembling a transient interdomain gas tunnel for ammonia delivery. Cryo‐EM snapshots reveal that GTP constitutes the ammonia tunnel and seals the GAT pocket^4^. Simultaneously, GTP binding stimulates glutamine hydrolysis by stabilizing the catalytic triad in the GAT domain^5^, ^6^. This dual role—substrate channeling and catalytic activation—eliminates futile cycles and ensures stoichiometric CTP production.

Each catalytic cycle couples CTP synthesis to a single GTP‐binding event, establishing a stoichiometric “timer” that prevents overaccumulation of CTP^4^. This contrasts sharply with hydrolytic GTPases like Ras, where GTP turnover drives irreversible transitions between active (GTP‐bound) and inactive (GDP‐bound) states^12^.

## EVOLUTIONARY CONSERVATION: TRACING GCP ORIGINS TO THE ARCHAEAN ERA

The discovery of conserved Phe/Trp/Arg residues in the GTP‐binding pockets of CTPS orthologs in archaeal (*Methanocaldococcus*), bacterial (*Escherichia coli*), and fungal (*Saccharomyces cerevisiae*) provides compelling evidence for the ancient origin of GCP motifs—structural and functional modules involving GTP coordination that are critical for enzymatic regulation^4^, ^13^. Phylogenetic analysis indicates these proteins originated over 2 billion years ago, predating the divergence of prokaryotes and eukaryotes. This timeline aligns with the Great Oxidation Event (~2.4 billion years ago), suggesting GCP motifs evolved as a metabolic innovation to address oxidative stress and nutrient scarcity in early Earth environments. Structural comparisons further demonstrate that the spatial arrangement of these residues—forming a triad critical for GTP coordination—is invariant across taxa, underscoring their functional indispensability.

GCP motifs are conserved functional units that integrate GTP binding, nucleotide sensing, and catalytic regulation^4^. Unlike canonical GTPases that dissipate energy via hydrolysis, GCP motifs utilize iterative GTP binding/dissociation to orchestrate catalytic cycles tightly coupled to cellular energy status.

GCP motifs are embedded not only in CTPS but also in enzymes central to nitrogen and carbon metabolism^6^. This suggests that GTP cycling evolved as a primordial strategy to optimize energy efficiency without compromising catalytic precision. The metabolic logic of GCP motifs becomes evident in extremophile proteomes, where their prevalence highlights their role as ancient metabolic adapters—enabling organisms to synchronize biosynthesis with energy fluxes in fluctuating environments.

## NON‐HYDROLYTIC REGULATION: REDEFINING METABOLIC EFFICIENCY

The non‐hydrolytic mechanism of GCPs represents a revolution in metabolic regulation, offering evolutionary and mechanistic advantages that transcend conventional GTPase systems. Unlike hydrolytic GTPases—which dissipate energy through irreversible GTP‐to‐GDP conversion—GCPs employ reversible GTP binding/dissociation to gate enzymatic activity, creating a dynamic equilibrium that optimizes energy use and catalytic precision (Table 1)^13^, ^14^. This mechanism not only preserves cellular energy reserves but also introduces novel layers of metabolic control, positioning GCPs as indispensable regulators of cellular homeostasis.

**Table 1:** Core differences between GTPase and GCPs.

Feature	GTPase	GCP
GTP metabolism	Hydrolyzing GTP to GDP (energy release)	Cycling GTP binding/dissociation (no hydrolysis)
Energy consumption	High (relying on hydrolysis for energy)	Low (relying on conformational changes)
Regulatory mechanism	Linear GEF/GAP‐mediated regulation	Bidirectional site coordination (positive/negative feedback)
Signal dynamics	Transient responses (seconds)	Sustained modulation (minutes)
Biological roles	Cell cycle progression, migration, and apoptosis	Metabolic homeostasis, stress tolerance, and pathogen survival

GEE/GAP, guanine nucleotide exchange factor (GEF)/GTPase‐activating protein (GAP).

### Energy preservation through reversible cycling

The core of GCP function lies in its ability to bypass the entropic cost of phosphate bond cleavage. Hydrolytic GTPases, such as Ras or Gα subunits, expend ~50 kJ/mol of energy per GTP hydrolysis event to drive conformational changes, with ~70% of this energy lost as heat^15^. In contrast, GCPs utilize non‐hydrolytic cycling to transiently stabilize active conformations, enabling sustained activity without ATP depletion. For instance, in *E. coli*, CTPS maintains nucleotide biosynthesis under anaerobic conditions by coupling GTP binding to GAT domain activation, a process requiring no ATP input^6^, ^16^, ^17^. The absence of catalytic Gln (Q61 in Ras) in *E. coli* CTPS—conserved from archaea to bacteria—confirms GCPs as an ancient energy‐saving strategy^13^, ^18^. This energy efficiency is critical for extremophiles like *Deinococcus radiodurans*, where GCP‐mediated pathways enable survival under oxidative stress by recycling nucleotide triphosphates without triggering ATP‐dependent stress responses^19^.

### Dynamic feedback and metabolic adaptability

GCPs act as “molecular rheostats”, translating GTP availability into catalytic output through reversible conformational gating. This feedback loop allows organisms to rapidly adjust biosynthetic flux in response to metabolic shifts. Such precision enables bacteria to reallocate resources within minutes, a trait essential for competition in nutrient‐limited environments.

### Noise reduction via spatial confinement

Hydrolytic systems are prone to futile cycles due to transient GTP binding events, leading to stochastic fluctuations in metabolic outputs. GCPs eliminate this noise through spatial segregation of intermediates. In CTPS, the GAT and synthase domains form a 20‐Å internal tunnel that shields intermediates like 4Pi‐UTP from hydrolytic enzymes, ensuring unidirectional flux^4^. This architectural precision minimizes off‐pathway reactions, achieving >95% of theoretical maximum—a level rarely observed in hydrolytic systems, where product inhibition reduces efficiency by 30%–50% in many cases.

## MECHANISTIC DRIVING FORCES BEHIND NON‐HYDROLYTIC GTP BINDING

### Electrostatic reorganization and charge stabilization

GTP binding induces a redistribution of charges within the nucleotide‐binding pocket^6^. For example, in CTPS, the GTP γ‐phosphate interacts with conserved basic residues, stabilizing a polarized charge network that drives conformational rearrangements. GTP binding may shift electron density toward the β‐phosphate, creating a partial positive charge that primes the enzyme for catalytic transitions.

### Conformational entropy and asymmetric binding

GTP binding restricts the conformational freedom of flexible loops, reducing entropy and enforcing a catalytically competent state^6^. This asymmetry prevents reverse transitions, as dissociation requires overcoming entropic penalties.

### Domain communication

In CTPS, GTP binding induces a helical twist in the GAT domain, propagating conformational changes to the catalytic triad via hydrogen‐bond networks.

### Strategies to avoid futile cycling

Possible strategies include cooperative binding and allosteric modulation. In metabolic filaments and cytoophidia, GTP binding to one protomer induces conformational changes in adjacent subunits, creating a cooperative network that amplifies directional signaling^20^. Additional considerations go to dynamic equilibrium and substrate channeling. In CTPS, the ammonia tunnel is only formed when GTP binds, ensuring NH₃ delivery and glutamine hydrolysis are kinetically linked^4^, ^5^, ^6^.

## THERAPEUTIC IMPLICATIONS: TARGETING GCP CYCLES IN PATHOGENS

The unique gating mechanism of GCPs presents a transformative opportunity for developing antimicrobial therapies with reduced off‐target effects. Pathogens such as *Mycobacterium tuberculosis* and *Plasmodium falciparum* exhibit strict dependence on GTP‐dependent enzymes like CTPS for nucleotide biosynthesis, creating a metabolic vulnerability. In contrast, human cells utilize alternative pathways for pyrimidine synthesis, enabling selective targeting of microbial GCP cycles without compromising host viability.

### Small‐molecule inhibitors

Competitive antagonists targeting GCPs’ GTP‐binding pockets can block conformational transitions essential for catalysis.

### Allosteric modulators

Exploiting intermediate‐dependent checkpoints offers a complementary approach. The 4Pi‐UTP intermediate, formed during CTP biosynthesis, induces a high‐affinity GTP‐binding conformation in CTPS^4^, ^5^, ^6^. Allosteric modulators binding to this intermediate—such as pyrimidine analogs with modified phosphate groups—destabilize the catalytic cycle by preventing GTP rebinding.

### CRISPR‐based disruption

CRISPR‐Cas9 systems may precisely edit residues critical for GTP dissociation. These strategies potentially leverage the evolutionary conservation of GCPs across pathogens. GCP targeting may circumvent antibiotic resistance mechanisms tied to traditional drug classes.

## CONCLUSION: A NEW FRONTIER IN ALLOSTERY

The discovery of GCPs as ancient allosteric regulators fundamentally reshapes our understanding of enzymatic control. By integrating non‐hydrolytic signal transduction with precision, GCPs challenge conventional paradigms of metabolic regulation, offering a unified framework to explain how life balances energy economy with catalytic fidelity. Their evolutionary conservation—from *Methanocaldococcus* to humans—highlights their role as primordial metabolic “adapters,” enabling organisms to thrive in fluctuating environments through reversible GTP‐dependent gating. Here, I underscore their dual identity as both metabolic sensors and actuators, capable of dynamically tuning biosynthetic fluxes in response to nucleotide triphosphate availability.

The mechanistic versatility of GCPs unlocks transformative applications across biotechnology and medicine. In synthetic biology, GCP‐inspired systems enable programmable biosynthesis platforms where metabolic flux is controlled by exogenous GTP pulses. These advancements hinge on decoding the “allosteric lexicon” of GCPs—a task accelerated by cryo‐EM and machine learning, which now resolve millisecond‐scale conformational changes and predict GTP‐binding hotspots.

Yet challenges persist. The structural heterogeneity of GCPs across taxa complicates drug design, while off‐target effects in host pathways (e.g., human CTPS isoforms) demand higher specificity. Emerging evidence reveals a hierarchical architecture for GCPs: (1) Metabolic filaments and cytoophidia formed by CTPS, as the prototype GCP1, represent functional superstructures that regulate nucleotide biosynthesis through allosteric channeling; (2) CTPS exhibits conserved filamentation mechanisms that modulate activity via nucleotide‐dependent conformational switches; (3) Phylogenetic analysis suggests unexplored GCP families (e.g., hypothetical GCPx) with distinct domain architectures, necessitating structural genomics to decode their evolutionary origins and functional niches^7^, ^8^, ^9^, ^10^, ^11^, ^20^.

Future breakthroughs will require interdisciplinary collaboration: combining evolutionary genomics to trace GCP origins, single‐molecule techniques to map allosteric networks, and AI‐driven platforms to optimize GCP‐based biocatalysts. By bridging ancient enzymology with modern engineering, GCP research heralds a metamorphosis—one where metabolic control is no longer constrained by hydrolytic energy waste, but reimagined through the lens of non‐hydrolytic allostery. This frontier promises not only to revolutionize biomedicine but also to redefine sustainability in industrial biotechnology, proving that the past holds the key to future innovation.

In summary, I herein introduce GCPs as a novel class of allosteric regulators that utilize iterative, non‐hydrolytic GTP binding/dissociation cycles to gate enzymatic transitions. This framework builds on my laboratory's prior investigations^4^, ^5^, including structural and evolutionary analyses of metabolic enzymes, and centers on cryo‐EM studies of CTPS, the rate‐limiting enzyme in de novo CTP biosynthesis. Our findings identify three sequential GTP‐dependent transitions governing CTP biosynthesis: (1) Catalytic Initiation Gate, where GTP dissociation occurs before substrate binding, enabling CTPS to adopt a catalytically competent conformation; (2) Intermediate‐Dependent GTP Recruitment, where 4Pi‐UTP formation stabilizes an allosteric GTP‐binding interface; (3) Ammonia Transfer Gate, where GTP directly participates in forming a transient internal tunnel for substrate channeling. This mechanistic framework positions GTP cycling as a kinetic checkpoint synchronizing glutamine hydrolysis with CTP synthesis. Phylogenetic analysis of GTP‐binding pockets across archaeal, bacterial, and fungal CTPS orthologs reveals conserved residues tracing GCP origins to >2 billion years ago. This non‐hydrolytic mechanism minimizes energy expenditure while enabling nutrient‐responsive metabolic flexibility, positioning GCPs as critical effectors of microbial resilience under stress. Collectively, GCPs redefine nucleotide‐driven regulation, offering a paradigm shift in metabolic engineering and antimicrobial strategy.
